# A Machine Learning-Based Prediction Platform for P-Glycoprotein Modulators and Its Validation by Molecular Docking

**DOI:** 10.3390/cells8101286

**Published:** 2019-10-21

**Authors:** Onat Kadioglu, Thomas Efferth

**Affiliations:** Department of Pharmaceutical Biology, Institute of Pharmacy and Biochemistry, Johannes Gutenberg University, 55128 Mainz, Germany; kadioglu@uni-mainz.de

**Keywords:** artificial intelligence, drug discovery, machine learning, molecular docking, multidrug resistance, P-glycoprotein

## Abstract

P-glycoprotein (P-gp) is an important determinant of multidrug resistance (MDR) because its overexpression is associated with increased efflux of various established chemotherapy drugs in many clinically resistant and refractory tumors. This leads to insufficient therapeutic targeting of tumor populations, representing a major drawback of cancer chemotherapy. Therefore, P-gp is a target for pharmacological inhibitors to overcome MDR. In the present study, we utilized machine learning strategies to establish a model for P-gp modulators to predict whether a given compound would behave as substrate or inhibitor of P-gp. Random forest feature selection algorithm-based leave-one-out random sampling was used. Testing the model with an external validation set revealed high performance scores. A P-gp modulator list of compounds from the ChEMBL database was used to test the performance, and predictions from both substrate and inhibitor classes were selected for the last step of validation with molecular docking. Predicted substrates revealed similar docking poses than that of doxorubicin, and predicted inhibitors revealed similar docking poses than that of the known P-gp inhibitor elacridar, implying the validity of the predictions. We conclude that the machine-learning approach introduced in this investigation may serve as a tool for the rapid detection of P-gp substrates and inhibitors in large chemical libraries.

## 1. Introduction

ATP-binding cassette (ABC) transporters are energy-dependent efflux pumps responsible for the active efflux of drugs, thereby reducing their intracellular concentration. Due to overexpression of ABC transporters in tumor cells, multidrug resistance (MDR) develops, which leads to the failure of chemotherapy with fatal consequences for cancer patients [1]. P-glycoprotein, being a well-known member among the ABC transporter family, is encoded by the *ABCB1*/*MDR1* gene. It is an important determinant of MDR [2,3,4] and upregulated in many clinically resistant and refractory tumors [5,6]. Its overexpression in tumor cells is associated with efficient extrusion of a large number of established anticancer drugs and natural cytotoxic products out of cancer cells, representing a major drawback of cancer chemotherapy [7]. Resistance is either inherently present or will be acquired during chemotherapy [8,9,10]. Hence, P-glycoprotein (P-gp) represents an important target to search for pharmacological inhibitors to overcome MDR [11]. Targeting P-gp to overcome MDR is of importance to achieve higher success rates for chemotherapy. The concept is to combine P-gp inhibitors with established chemotherapy drugs to resensitize tumors [12,13,14,15].

Machine learning and artificial intelligence are recently acquiring increasing interest in the area of drug discovery [16,17,18] because these methods have an enormous potential to speed up the preclinical development processes at minimal costs. For this purpose, we utilized a machine learning strategy in order to establish a prediction platform that allows to predict whether a given compound behaves as a substrate or an inhibitor of P-gp. 

Available natural compound databases serve as an invaluable source to identify novel lead compounds that possess activity against certain diseases or disorders by focusing on particular target biomarker proteins. As a majority of established anticancer drugs are of natural origin [19], natural products may serve as lead compounds for derivatization to obtain novel chemical entities with improved pharmacological features. Analyses of the interaction between the compounds and the target protein with molecular docking provide clues about the possible binding mode and binding energy, as we reported before [11,20,21]. Selecting P-gp as target protein, the interaction of test compounds can be compared with that of known P-gp inhibitors, such as verapamil, valspodar, tariquidar, or elacridar, in order to assess their binding properties, docking poses, and binding energies. In those cases, where the test compounds yielded by using the P-gp modulator prediction platform possess similar docking poses and comparable binding energies as known inhibitors, it could be concluded that these compounds may be potential P-gp inhibitors. 

In the present study, we used machine learning strategies to establish such a P-gp modulator prediction platform for compounds by using defined chemical descriptors to predict whether a given compound can behave as a substrate or an inhibitor of P-gp. Selected compounds from inhibitor or substrate classes were subjected to molecular docking for further verification and compared with known P-gp inhibitors and substrates.

## 2. Material and Methods

### 2.1. Preparation of Compound List and Calculation of Chemical Descriptors

For the P-gp modulator/non-modulator prediction model, a compound list with modulators and non-modulators from Broccatelli et al. [22] was used. Compounds for learning and validation steps were randomly selected. Thirty-two modulator and thirty-two non-modulator compounds were used for the learning step, while 16 modulator and 16 non-modulator substances were used for the validation step (Table 1). For the P-gp inhibitor/substrate prediction model, a list of P-gp substrates and inhibitors was prepared by referring to the literature [23], yielding a total of 60 compounds (34 inhibitors, 26 substrates). Again, compounds for learning and validation steps were randomly selected. Forty compounds (20 inhibitors, 20 substrates) were used for learning and model establishment. The remaining 20 compounds (14 inhibitors, 6 substrates) were used for the external validation step (Table 2). 

Data Warrior software is a multipurpose chemistry data visualization and data analysis program that calculates various molecular descriptors and properties for a given set of compounds. It was used to calculate the chemical descriptors as previously reported [24,25]. After calculation of the 32 chemical descriptors, correlation coefficients between descriptors and correlation of the descriptors with the P-gp modulator category (substrate or inhibitor) were determined using SPSS statistics software version 23.0.0.3 (IBM, Armonk, NY: IBM Corp, USA). If the correlation coefficient between the P-gp modulator category (substrate or inhibitor) and a certain descriptor was below 0.1, this descriptor was omitted. Only descriptors correlating with the P-gp modulator (substrate or inhibitor) category above 0.1 were selected for further processing. As a next step, descriptors having a pairwise correlation coefficient to the P-gp modulator category lower than 0.9 were excluded [26]. By this strategy, relevant descriptors without an issue of over-fitting can be selected.

### 2.2. P-Glycoprotein Modulator Prediction Model Establishment

**Figure 1:**
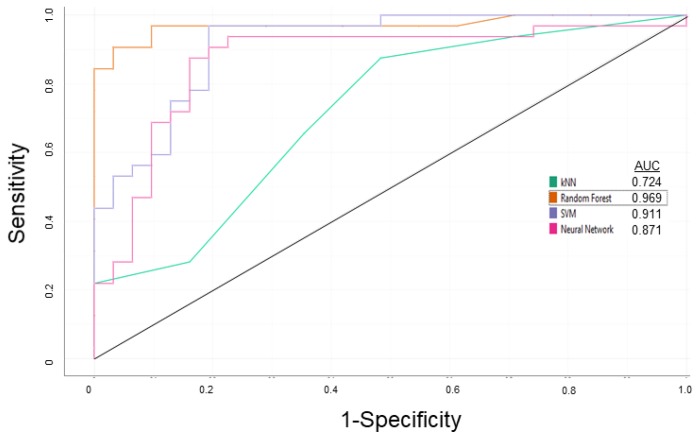
Receiver operating characteristic (ROC) curves of k Nearest Neighboring (kNN), Neural Network, Random Forest (RF), and Support Vector Machine (SVM) classification algorithms based on random leave-one-out sampling for the P-gp modulator/non-modulator prediction model for the learning step.

At first, a model, which can predict whether a given compound is a P-gp modulator, was built by using the compound list from Broccatelli et al. [22] After applying the descriptor selection criteria by considering the relevancy and over-fitting issues, “logP”, “H-donors”, “polar surface area”, “ligand efficiency dependent lipophilicity”, “molecular complexity”, “stereo centers”, “rotatable bonds”, “rings closures”, “aromatic rings”, “sp-3 atoms”, “amides”, “amines”, “alkyl-amines, ”and “basic nitrogens” were considered for the preparation of the P-gp modulator/non-modulator prediction model. Various classification algorithms with the leave-one-out random sampling method were tested, i.e., k-Nearest Neighboring (kNN), Neural Network, Random Forest (RF), and Support Vector Machine (SVM). Receiver operating characteristic (ROC) curves are depicted in Figure 1. The receiver operating characteristic (ROC) curve plotted the true positive rate (= sensitivity) against the false positive rate (= 1-specificity). The RF algorithm performed better than the other classification algorithms both in learning and validation steps. The overall performance for the established model based on RF algorithm is summarized in Table 3. The establishment of the P-gp modulator/non-modulator and P-gp inhibitor/substrate prediction models were performed by using the machine learning software Orange (Ljubljana, Slovenia) [27]. 

**Figure 2:**
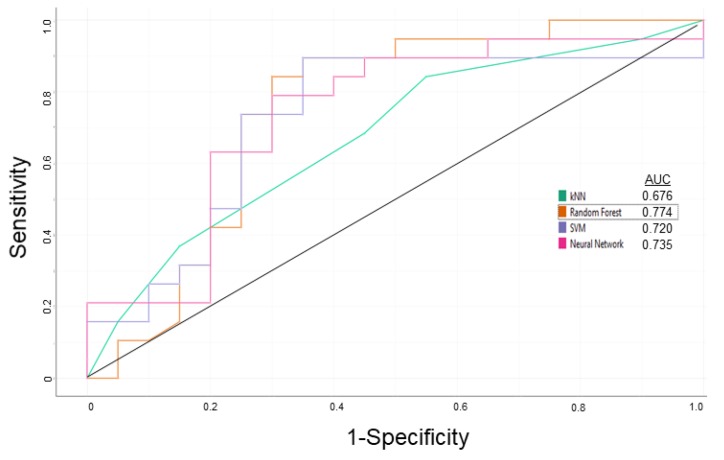
ROC curves of kNN, Neural Network, RF, and SVM classification algorithms based on random leave-one-out sampling for the P-gp inhibitor/substrate prediction model for the learning step.

After applying the descriptor selection criteria by considering the relevancy and over-fitting issues, “logP”, “total surface area”, “shape index”, “molecular flexibility”, “rotatable bonds”, “aromatic rings”, “aromatic atoms”, “aromatic nitrogens”, “basic nitrogens”, “symmetric atoms”, and “acidic oxygens” were considered for P-gp inhibitor/substrate prediction model preparation. Various classification algorithms with the leave-one-out random sampling method were tested, i.e., kNN, Neural Network, RF, and SVM. The ROC curves are depicted in Figure 2. The RF algorithm performed better than the other classification algorithms. The overall performance for the established model is summarized in Table 4.

In order to evaluate the model performance further and select potential inhibitors, a P-gp modulator compound list consisting of 643 compounds from ChEMBL was used. 

### 2.3. Molecular Docking

The recently published human P-gp structure was used (nanodisc reconstituted in complex with UIC2 fab and paclitaxel at the drug-binding pocket, PDB ID: 6QEX, in the absence of a lipid bilayer) [28]. The Fab chains were deleted. The bound ligands marked as “HETATM” including taxol were also deleted from the PDB structure file in order to prevent interference with molecular docking. The preparation of the final receptor structure as “.pdbqt” file was performed with Autodock tools 1.5.7. Selected compounds from inhibitor and substrate classes have been subjected to an automated and comprising molecular docking campaign by using the high-performance supercomputer MOGON (Johannes Gutenberg University, Mainz). Compound flexibilities were taken into account and a rigid receptor structure was used. At first, three independent screening of all 643 compounds from ChEMBL with Autodock Vina algorithm was performed by focusing on the drug-binding pocket of P-gp, where the majority of the known inhibitors and substrates bind to. The grid parameters are listed in Table 5. 

Afterward, the top 20 compounds in terms of binding energy yielded from both inhibitor and substrate predictions were selected for molecular docking. Each molecular docking was based on three independent dockings each consisting of 2,500,000 calculations. This means that each data point represents the mean value of 7,500,000 individual MOGON-based calculations. The Autodock 4 algorithm was used for defined molecular docking calculations on the drug-binding pocket of P-gp as described before [11], and Visual Molecular Dynamics (VMD) software (Theoretical and Computational Biophysics group at the Beckman Institute, University of Illinois at Urbana-Champaign) was used for the visualization of the docking poses. Estimated inhibition constants were calculated by the Autodock algorithm with the equation:

(1) $$Ki=\exp\left(\frac{\Delta G}{R*T}\right)$$

*Ki* (M)
Δ*G* (cal/mol) = 1000 * LBE (lowest binding energy, kcal/mol)
*R* (cal/mol-K): gas constant, 1.986 cal/mol-K
*T* (K): room temperature, 298 K

### 2.4. Boxplot Analysis

The distribution of the values for the descriptors used for the P-gp inhibitor/substrate prediction model and the comparison for the predicted inhibitors and substrates among the ChEMBL P-gp modulator list were subjected to Boxplot analysis using Microsoft Excel 2019 (Microsoft, USA). Statistical significances were evaluated by the t-test (two-tailed, two-sample unequal variance).

## 3. Results

### 3.1. P-glycoprotein Modulator Predictions

The P-gp modulator/non-modulator prediction model was evaluated with the validation set as mentioned in the corresponding method part. The RF algorithm reached 0.938 for all parameters. The ChEMBL P-gp modulator list of 643 compounds was tested, and 641 out of 643 substances were correctly predicted as modulators. 

The P-gp inhibitor/substrate prediction model with the ChEMBL P-gp modulator list of 643 compounds was evaluated. A total of 493 substances were predicted as inhibitors, and 150 compounds were predicted as substrates. Subjecting all compounds to Autodock Vina screening allowed to rank them according to their binding energies. The top 20 inhibitor predictions with strong interaction to P-gp are shown in Table 6. These inhibitors were selected for subsequent molecular docking. The top 20 substrate predictions with strong interaction to P-gp are shown in Table 7. These substrates were also selected substances for subsequent molecular docking. The complete predictions for all 493 inhibitors together with their binding affinities to P-gp are shown in Supplementary Table S1, while all predictions for the 150 substrates and their affinities to P-gp are listed in Supplementary Table S2. The average lowest binding energy (LBE) was -8.155 for the inhibitors and -9.289 for the substrates. 

Among the 493 inhibitor compounds were 117 natural products (= 23.7%), while all other compounds were of synthetic origin (Supplementary Table S1). The proportion of natural products was higher among the predicted P-gp substrates (69/150 = 46%) (Supplementary Table S2). This trend was even more apparent if we focused on the top 20 inhibitor or substrate compounds only (Table 6 and Table 7). Here, 2/20 (= 10%) were predicted inhibitors, but 11/20 (= 55%) were predicted substrates, indicating that P-glycoprotein may expel natural xenobiotics from cells with higher probability.

### 3.2. Molecular Docking

After running the prediction model on the P-gp modulator list from ChEMBL and the Autodock VINA screening, the top 20 compounds from the inhibitor class and the top 20 compounds from the substrate class were selected for molecular docking analyses on human P-gp. The lowest binding energies (LBE) and predicted inhibition constants are listed in Table 8 for the inhibitors and Table 9 for the substrates. 

**Figure 3:**
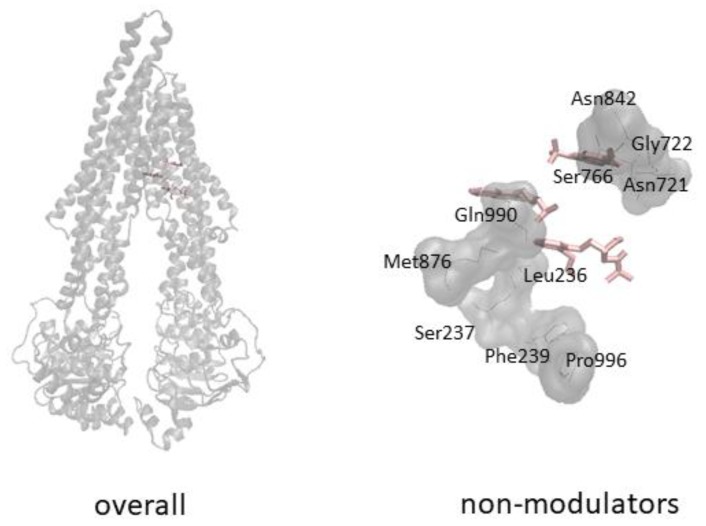
Molecular docking results for selected non-modulators (pink).

The negative control compounds (oxprenolol, promazine, riluzole) revealed weaker interaction with P-gp (Table 10) and slightly different docking pose as well (Figure 3). 

**Figure 4:**
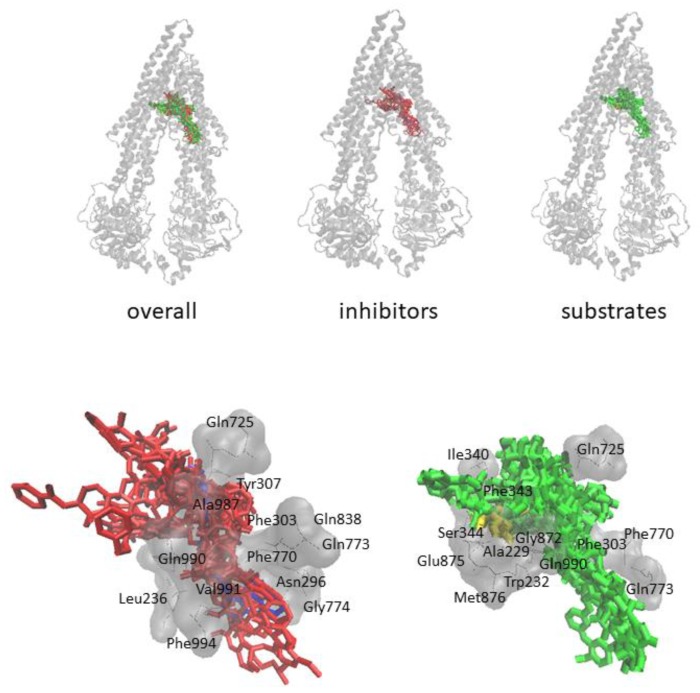
Molecular docking results for selected inhibitors (red) and substrates (green) yielded from the P-gp inhibitor/substrate prediction model. Elacridar (blue) and doxorubicin (yellow) were selected as control drugs.

As can be seen in Figure 4, the predicted inhibitors possessed similar docking poses as elacridar at the drug-binding pocket of P-gp. Similar results were observed for the substrates: The predicted substrates revealed similar docking poses as doxorubicin. Hence, these results validated the precision and reliability of the model.

Predicted inhibitors and substrates interact with P-gp significantly stronger than the negative control compounds. This is clear both from the binding energies and predicted inhibition constants. Binding energies of non-modulators are within −5.380 (piluzole) to −6.933 (promazine) kcal/mol and the predicted inhibition constants are within 8.273–114.080 µM, whereas binding energies for the predicted substrates are within −7.337 (vindoline) to −12.500 (latilagescene G) and for the predicted inhibitors −8.900 (3-methylcholanthrene) to −13.537 (karavoate P). Predicted inhibition constants for the predicted substrates are within 0.001–4.363 and for the predicted inhibitors 0.0002–0.300 µM. Docking pose of the negative control compounds differs from that of inhibitors and substrates. Overall, it can be speculated that the predicted inhibitors interact with P-gp stronger than the predicted substrates and the non-modulators are making weak interactions with P-gp and they bind to a different site. 

**Figure 5:**
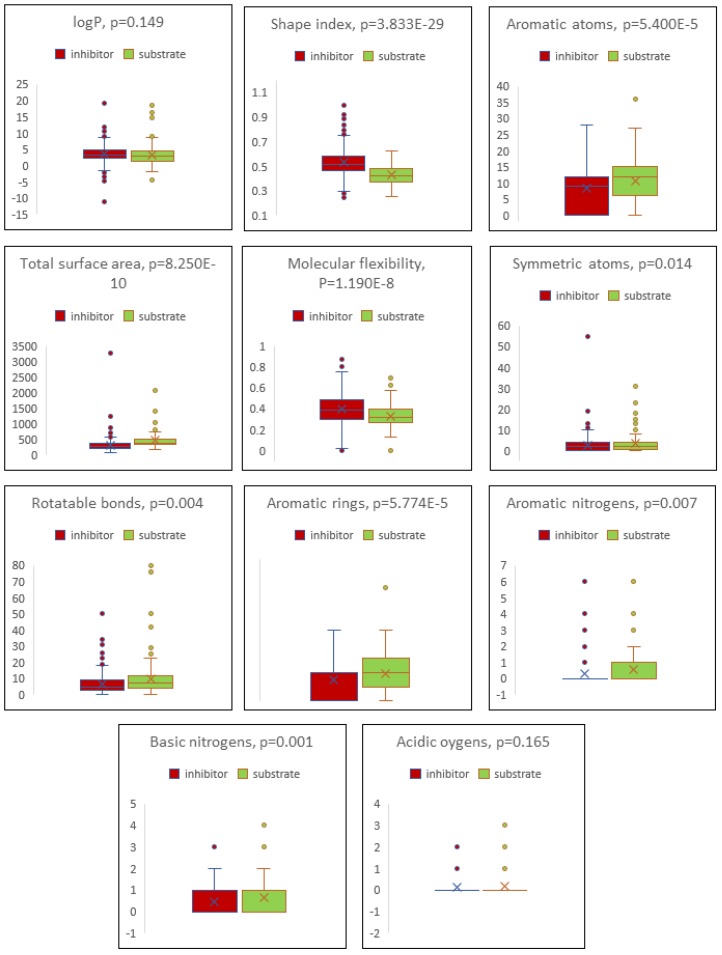
Boxplot analysis of the descriptors used for the model and comparison of the predicted inhibitors and substrates.

The distribution of the values for the descriptors used to build the model and the comparison for the predicted inhibitors and substrates in terms of those descriptor values were performed with Boxplot analysis. As can be seen from Figure 5, the inhibitors revealed significantly different values for all descriptors except logP and acidic oxygens. The average values of descriptors for inhibitors and substrates are listed in Table 11.

## 4. Discussion

In the present study, we utilized machine learning methods based on leave-one-out random sampling in order to develop a P-gp modulator prediction platform by using chemical descriptors. The main focus was to predict whether a given compound can behave as substrate or inhibitor of P-gp. The RF classification algorithm (AUC:0.774) outperformed the other tested algorithms (kNN—0.676, Neural Network—0.745, SVM—0.720). Performance scores for the external validation set were even higher than the learning set with better sensitivity (0.786 vs. 0.750), specificity (0.833 vs. 0.700), overall prediction accuracy (0.800 vs. 0.725), and precision (0.917 vs. 0.714). Further testing with the P-gp modulator list from ChEMBL yielded promising results with accurate predictions. Four compounds from inhibitor and four compounds from substrate prediction list were selected for molecular docking analyses. Validations with molecular docking on a recently released human P-gp structure were performed in terms of binding energy and docking poses by including known inhibitor (elacridar) and substrate (doxorubicin) as controls. Curcumin, miconazole, tacrolimus, and venlafaxine revealed a similar docking pose at the drug-binding pocket of P-gp with comparable binding energies with that of elacridar. MK-3207, rifampin, vindoline, and voacamine revealed similar docking poses and comparable binding energy with those of doxorubicin. Overall, the precision and reliability of the model were further confirmed. 

Machine learning and artificial intelligence attracted increasing interest in the drug discovery area [18,29,30], and utilizing these methods possess great potential for drug discovery, as they save time and costs during the preclinical steps. The RF algorithm depends on multiple decision trees that are built based on the training data, and a majority voting scheme is used to make classification or regression predictions [31]. RF application to drug discovery has been recently reported, and it outperformed other algorithms such as SVM and NN in terms of feature selection [32]. 

There are various studies in the literature that utilized machine-learning strategies focusing on P-gp. One study pointed out a P-gp substrate prediction model based on RF algorithm to estimate transport potential for central nervous system drugs, accuracy lies between 0.713 and 0.846 whereas precision is between 0.633 and 0.777 [33]. Our P-gp modulator prediction model involves an accuracy of 0.953 for the learning set and 0.938 for the validation set, and our P-gp inhibitor prediction model has an accuracy value of 0.725 for the learning set and 0.800 for the validation set. In terms of precision, our models also perform better. Modulator prediction model involves a precision of 0.968 for the learning set and 0.938 for the validation set. Inhibitor prediction model has a 0.714 precision for the learning set and 0.917 for the validation set. Similarly, a P-gp substrate efflux ratio prediction model has been recently reported based on SVM algorithm [34]. The affinities of flavonoids to P-gp have been evaluated with an SVM-based model and a high correlation with the experimental data has been achieved [35]. Another study involving P-gp inhibitor prediction was performed for chalcone derivatives and selected inhibitor candidates were analyzed in terms of their docking pose on a homology model of human P-gp [36]. The prediction of blood–brain barrier permeability mechanism of central nervous system drugs has been utilized with an SVM-based model [37]. Binding pattern prediction based on pharmacophore ensemble/SVM method for potential P-gp inhibitors was also recently reported [38]. Another SVM-based model coupled with molecular docking aimed to predict whether a given compound may act as P-gp substrate, the accuracy lies between 0.750 and 0.800, specificity between 0.750 and 0.810, and sensitivity between 0.740 and 0.790 [39]. Our modulator prediction model outperforms that model in all those parameters. Our inhibitor prediction model outperforms in the validation set. Similarly, in 2004, SVM-based P-gp substrate prediction model was reported; sensitivity was 0.812, specificity was 0.792, and accuracy was 0.794 [40]. Our modulator prediction model outperforms that model in all those parameters. Our inhibitor prediction model outperforms in the validation set for the specificity and accuracy parameters. In general, these previously published studies have certain disadvantages, e.g., low performance scores in terms of prediction, focusing on only P-gp substrate prediction or molecular docking with homology models but not crystal structures. Our model is superior compared to the previously published studies for several reasons. It is based on leave-one-out random sampling RF algorithm, focused on both natural as well as synthetic compounds, has high sensitivity, specificity, predictive accuracy, and precision to predict at first P-gp modulator/non-modulator and as a next step to predict P-gp substrate/inhibitor depending on various chemical descriptors, and it was coupled with molecular docking using the recently released crystal structure of human P-gp. The fact that predictions on the P-gp modulator list of compounds from ChEMBL was validated with accurate molecular docking results was also advantageous for our model. Furthermore, after the initial compound screening, selected inhibitors revealed similar docking poses as elacridar (as positive control for an inhibitor) and selected substrates revealed similar docking poses as doxorubicin (as positive control for a substrate). Non-modulators have significantly weaker interaction with P-gp and they bind to a slightly different position. Overall, those observations provide further clues for the reliability of the prediction model. 

Selected inhibitors and substrates after the virtual screening are supported by literature; astemizole [41], cryptotanshinone [42], dihydrocytochalasin B [43], jolkinol B [44], latilagascenes D [45], lonafarnib [46], tariquidar [12], zosuquidar [47], acetyldigitoxin [48], bromocriptine [49], candesartan cilexetil [50], cepharanthin [51], cytochalasin E [52], digitoxin [53], digoxin [54], dihydroergosrictine [55], dofequidar [56], ergocristine [55], irinotecan [57], latilagascenes E [45], MK-3207 [58], paclitaxel [59], vindoline [60].

Many cancer types involve P-gp overexpression, which is associated with increased efflux of established anticancer drugs and natural cytotoxic products out of cancer cells. This phenomenon represents a major drawback of cancer chemotherapy with limitations in killing tumor populations due to MDR [61,62]. P-gp overexpression is indeed one of the main reasons for MDR and thus inadequate chemotherapy success rate. Targeting P-gp is critical to achieve high success rates for chemotherapy, therefore, identification of novel P-gp inhibitors is critical in that regard. 

Our prediction platform for P-gp modulators facilitates to predict whether a given compound can behave as a substrate or an inhibitor of P-gp. The selection of potential inhibitors can be further validated by molecular docking and the comparison of the binding energy and docking pose with those of known P-gp inhibitors. As a next step in the future, our model may be helpful to identify potential novel P-gp inhibitors and to develop effective chemotherapy strategies involving combination therapy with targeted chemotherapy drugs and identified P-gp inhibitors. 

## 5. Conclusion

In the present study, we established P-gp modulator/non-modulator and inhibitor/substrate prediction models based on the RF algorithm and leave-one-out random sampling. Validation with molecular docking was performed. The identification of novel P-gp inhibitors is critical to overcome MDR and to achieve better chemotherapy strategies. This model can predict whether a given compound can behave as substrate or inhibitor of P-gp, and will be, thus, helpful to identify potential P-gp inhibitors. 

## Figures and Tables

**Table 1:** Compounds selected for learning and external validation for the P-glycoprotein (P-gp) modulator/non-modulator prediction model.

Learning Set				External Validation Set	
Compound	Category	Compound	Category	Compound	Category
Escitalopram	Modulator	Hydroxyzine	Non-modulator	Terfenadine	Modulator
Simvastatin acid	Modulator	Oxybutynin	Non-modulator	Prazosin	Modulator
Neostigmine	Modulator	Ethosuximide	Non-modulator	Prednisone	Modulator
Zolmitriptan	Modulator	Warfarin	Non-modulator	Chloroquine	Modulator
Atomoxetine	Modulator	Mexilitene	Non-modulator	Lopinavir	Modulator
Methysergide	Modulator	Sulpiride	Non-modulator	Prednisolone	Modulator
Famciclovir	Modulator	Thiopental	Non-modulator	Vincristine	Modulator
Lovastatin acid	Modulator	Lamotrigine	Non-modulator	Sertraline	Modulator
Darifenacin	Modulator	Diphenhydramine	Non-modulator	Loperamide	Modulator
Paliperidone	Modulator	Enoxacin	Non-modulator	Etoposide	Modulator
Trospium	Modulator	Methylphenidate	Non-modulator	Indinavir	Modulator
Aprepitant	Modulator	Itraconazole	Non-modulator	Dipyridamole	Modulator
Apomorphine	Modulator	Nortriptyline	Non-modulator	Mitoxantrone	Modulator
Cetirizine	Modulator	Galantamine	Non-modulator	Cimetidine	Modulator
Cyclosporin A	Modulator	Ramelteon	Non-modulator	Bromocriptine	Modulator
Labetalol	Modulator	Rivastigmine	Non-modulator	Reserpine	Modulator
Amisulpride	Modulator	Ropivacaine	Non-modulator	Oxprenolol	Non-modulator
5-Hydroxymethyl tolterodine	Modulator	Zonisamide	Non-modulator	Alprazolam	Non-modulator
Cabergoline	Modulator	Zolpidem	Non-modulator	Oxcarbazepine	Non-modulator
Ximelagatran	Modulator	Sulfasalazine	Non-modulator	Tolterodine	Non-modulator
Hoechst 33342	Modulator	Metoclopramide	Non-modulator	Zaleplon	Non-modulator
Rhodamine 123	Modulator	Nalmefene	Non-modulator	Cyclobenzaprine	Non-modulator
Actinomycin D	Modulator	Oxycodone	Non-modulator	Nimodipine	Non-modulator
Olanzapine	Modulator	Topiramate	Non-modulator	Riluzole	Non-modulator
Ranitidine	Modulator	Hydrocodone	Non-modulator	Tiagabine	Non-modulator
Astemizole	Modulator	Rosuvastatin	Non-modulator	Nalbuphine	Non-modulator
Verapamil	Modulator	Tropisetron	Non-modulator	Duloxetine	Non-modulator
Ziprasidone	Modulator	Varenicline	Non-modulator	Pravastatin acid	Non-modulator
Chlorpromazine	Modulator	Clemastine	Non-modulator	Promazine	Non-modulator
Clozapine	Modulator	Clonazepam	Non-modulator	Bromazepam	Non-modulator
Trimethoprim	Modulator	Ropinirole	Non-modulator	Lorazepam	Non-modulator
Paroxetine	Modulator	Solifenacin	Non-modulator	Mirtazapine	Non-modulator

**Table 2:** Compounds selected for learning and external validation for the P-gp inhibitor/substrate prediction model.

Learning Set				External Validation Set			
Compound	Category	Compound	Category	Compound	Category	Compound	Category
Ginsenoside	Inhibitor	Epirubicin	Substrate	Agosterol	Inhibitor	Colchicin	Substrate
Laniquidar	Inhibitor	Etoposide	Substrate	Amiodarone	Inhibitor	Dexamethazone	Substrate
Loratidine	Inhibitor	Fexofenadine	Substrate	Amorinin	Inhibitor	Digoxin	Substrate
Mibefradil	Inhibitor	Hoechst 33342	Substrate	Apigenin	Inhibitor	Docetaxel	Substrate
Naringenin	Inhibitor	Idarubicin	Substrate	Atorvastatin	Inhibitor	Doxorubicin	Substrate
Pgp-4008	Inhibitor	Irinotecan	Substrate	Atovaquone	Inhibitor	Daunorubicin	Substrate
Phloretin	Inhibitor	Kaempferol	Substrate	Biochanin	Inhibitor		
Quercetin	Inhibitor	Loperamide	Substrate	Biricodar	Inhibitor		
Quinine	Inhibitor	Mitomycin	Substrate	Catechin	Inhibitor		
Rotenone	Inhibitor	Mitoxantrone	Substrate	Cefoperazone	Inhibitor		
Sakuranetin	Inhibitor	Ondansetron	Substrate	Chrysine	Inhibitor		
Sertraline	Inhibitor	Paclitaxel	Substrate	Cyclosporine	Inhibitor		
Sinensetin	Inhibitor	Procyanidin B2	Substrate	Diltiazem	Inhibitor		
Stigmasterol	Inhibitor	Rhodamine 123	Substrate	Elacridar	Inhibitor		
Syringaresinol	Inhibitor	Tenoposide	Substrate				
Tamoxifen	Inhibitor	Topotecan	Substrate				
Tariquidar	Inhibitor	Vinblastine	Substrate				
Valspodar	Inhibitor	Vincristine	Substrate				
Verapamil	Inhibitor	Vindesine	Substrate				
Zosuquidar	Inhibitor	Vinorelbine	Substrate				

**Table 3:** Performance of the P-gp modulator/non-modulator prediction model based on the RF classifier algorithm.

Steps	Sensitivity	Specificity	Overall Predictive Accuracy	Precision
**Learning**	0.938	0.969	0.953	0.968
**External Validation**	0.938	0.938	0.938	0.938

**Table 4:** Performance of the P-gp inhibitor/substrate prediction model based on the RF classifier algorithm.

Steps	Sensitivity	Specificity	Overall Predictive Accuracy	Precision
**Learning**	0.750	0.700	0.725	0.714
**External Validation**	0.786	0.833	0.800	0.917

**Table 5:** Grid parameters for molecular docking analyses on human P-gp.

	x	y	z
**Number of Points**	126	98	116
**Grid Center**	168.614	166.372	162.000
**Grid Spacing (Å)**	0.375		

**Table 6:** Prediction of the top 20 P-gp inhibitors identified by the RF classification algorithm using the ChEMBL P-gp modulator list of 493 compounds. The results were validated by determining the binding affinities using Autodock VINA.

Name	ChEMBL ID	Inhibitor Probability	Class	VINA LBE (kcal/mol)
Karavoate P	CHEMBL1641677	0.849	Synthetic	−12.200 ± 1.212
Tribenzoylbalsaminol F	CHEMBL1928854	0.549	Synthetic	−12.033 ± 0.896
Zosuquidar	CHEMBL444172	0.513	Synthetic	−11.967 ± 0.058
Latilagascenes D	CHEMBL435917	0.566	Synthetic	−11.700 ± 0.001
Dihydrocytochalasin B	CHEMBL2074735	0.513	Synthetic	−11.367 ± 0.231
Jolkinoate I	CHEMBL2315618	0.593	Synthetic	−11.300 ± <0.001
Karavoate K	CHEMBL1641672	0.849	Synthetic	−11.267 ± 0.493
Fanchinin	CHEMBL176045	0.586	Synthetic	−11.233 ± 0.208
Latilagascene I	CHEMBL511018	0.586	Synthetic	−11.167 ± 0.058
Karavoate L	CHEMBL1641673	0.766	Synthetic	−11.133 ± 0.808
3-Methylcholanthrene	CHEMBL40583	0.788	Synthetic	−11.100 ± <0.001
Lonafarnib	CHEMBL298734	0.567	Synthetic	−11.000 ± <0.001
Karavoate N	CHEMBL1641675	0.666	Synthetic	−10.933 ± 0.058
Tariquidar	CHEMBL348475	0.619	Synthetic	−10.933 ± 0.404
Pimozide	CHEMBL1423	0.517	Synthetic	−10.900 ± 0.100
Karavoate I	CHEMBL1641670	0.766	Synthetic	−10.767 ± 0.058
Cryptotanshinone	CHEMBL187460	0.663	Natural	−10.700 ± <0.001
Jolkinol B	CHEMBL489265	0.577	Synthetic	−10.700 ± <0.001
Astemizole	CHEMBL296419	0.617	Synthetic	−10.667 ± 0.115
Metergoline	CHEMBL19215	0.732	Natural	−10.600 ± <0.001

**Table 7:** Prediction of P-gp substrates identified by the RF classification algorithm using the ChEMBL P-gp modulator list of 150 compounds. The results were validated by determining the binding affinities using Autodock VINA.

Name	ChEMBL ID	Substrate probability	Class	VINA LBE (kcal/mol)
Vindoline	CHEMBL526546	0.771	Synthetic	−15.000 ± <0.001
Cepharanthin	CHEMBL2074948	0.614	Natural	−12.600 ± <0.001
Latilagascene G	CHEMBL448193	0.514	Synthetic	−12.300 ± <0.001
Mk3207	CHEMBL1910936	0.733	Synthetic	−12.167 ± 0.058
Ergocristine	CHEMBL446315	0.767	Natural	−12.067 ± 0.058
Cytochalasin E	CHEMBL494856	0.6	Natural	−11.800 ± <0.001
Jolkinoate L	CHEMBL2315621	0.567	Synthetic	−11.533 ± 0.058
Irinotecan	CHEMBL481	0.967	Natural	−11.400 ± 0.819
Latilagascenes E	CHEMBL373511	0.614	Synthetic	−11.367 ± 0.116
Dofequidar	CHEMBL65067	0.583	Synthetic	−11.300 ± 0.001
Acetyldigoxin	CHEMBL2074725	0.708	Natural	−11.233 ± 0.808
Dihydroergocristine	CHEMBL601773	0.767	Natural	−11.133 ± 0.666
Telcagepant	CHEMBL236593	0.517	Synthetic	−11.067 ± 0.058
Ergotamine	CHEMBL442	0.8	Natural	−10.933 ± 0.058
Candesartan Cilexetil	CHEMBL1014	0.567	Synthetic	−10.900 ± 0.200
Digoxin	CHEMBL1751	0.708	Natural	−10.833 ± 1.097
Bromocriptine	CHEMBL493	0.767	Natural	−10.800 ± 0.100
Itrazole	CHEMBL64391	0.564	Synthetic	−10.700 ± 0.436
Digitoxin	CHEMBL254219	0.725	Natural	−10.667 ± 0.462
Paclitaxel	CHEMBL428647	0.808	Natural	−10.633 ± 0.462

**Table 8:** Lowest binding energies (LBE) and predicted inhibition constants obtained by molecular docking of the top 20 P-gp inhibitors.

P-gp Inhibitor	AutoDock LBE (kcal/mol)	Predicted Inhibition Constant (µM)
3-Methylcholanthrene	−8.900 ± 0.001	0.300 ± <0.001
Astemizole	−9.693 ± 0.047	0.079 ± 0.007
Cryptotanshinone	−9.010 ± 0.001	0.251 ± <0.001
Dihydrocytochalasin B	−10.460 ± 0.020	0.0212 ± 0.001
Fanchinin	−9.937 ± 0.067	0.0522 ± 0.006
Jolkinoate I	−10.440 ± 0.200	0.0232 ± 0.008
Jolkinol B	−10.250 ± 0.044	0.0307 ± 0.002
Karavoate I	−12.310 ± 0.235	0.001 ± <0.001
Karavoate K	−12.330 ± 0.213	0.001 ± <0.001
Karavoate L	−12.807 ± 0.200	0.0004 ± <0.001
Karavoate N	−12.160 ± 0.560	0.002 ± 0.001
Karavoate P	−13.537 ± 0.605	0.0002 ± <0.001
Latilagascene I	−11.147 ± 0.561	0.009 ± 0.009
Latilagascenes D	−12.220 ± 0.370	0.001 ± 0.001
Lonafarnib	−11.433 ± 0.087	0.004 ± 0.001
Metergoline	−9.737 ± 0.029	0.073 ± 0.004
Pimozide	−10.220 ± 0.324	0.031 ± 0.025
Tariquidar	−11.273 ± 0.274	0.006 ± 0.002
Tribenzoylbalsaminol F	−12.403 ± 0.118	0.001 ± <0.001
Zosuquidar	−11.257 ± 0.361	0.006 ± 0.004
Elacridar (positive control)	−11.093 ± 0.361	0.008 ± 0.004

**Table 9:** Lowest binding energies (LBE) and predicted inhibition constants obtained by molecular docking of the top 20 P-gp substrates.

P-gp substrate	AutoDock LBE (kcal/mol)	Predicted Inhibition Constant (µM)
Acetyldigoxin	−11.767 ± 0.480	0.003 ± 0.002
Bromocriptine	−12.360 ± 1.02	0.002 ± 0.001
Candesartan Cilexetil	−11.153 ± 0.370	0.007 ± 0.004
Cepharanthin	−10.753 ± 0.006	0.013 ± <0.001
Cytochalasin E	−10.957 ± 0.006	0.093 ± 0.001
Digitoxin	−11.390 ± 0.517	0.006 ± 0.004
Digoxin	−11.500 ± 0.151	0.004 ± 0.001
Dihydroergocristine	−11.670 ± 0.056	0.003 ± <0.001
Dofequidar	−10.970 ± 0.351	0.010 ± 0.006
Ergocristine	−12.407 ± 0.012	0.001 ± <0.001
Ergotamine	−11.227 ± 0.150	0.006 ± 0.001
Irinotecan	−11.380 ± 0.020	0.005 ± <0.001
Itrazole	−10.843 ± 0.186	0.012 ± 0.003
Jolkinoate L	−10.643 ± 0.681	0.022 ± 0.016
Latilagascenes E	−11.770 ± 0.185	0.002 ± 0.001
Latilagescene G	−12.500 ± 0.316	0.001 ± <0.001
Mk-3207	−11.650 ± 0.020	0.003 ± <0.001
Paclitaxel	−9.607 ± 0.359	0.103 ± 0.065
Telcagepant	−9.333 ± 0.021	0.144 ± 0.005
Vindoline	−7.337 ± 0.211	4.363 ± 1.389
Doxorubicin (positive control)	−11.070 ± 0.135	0.008 ± 0.002

**Table 10:** Lowest binding energies (LBE) and predicted inhibition constants obtained by molecular docking of the non-modulators.

P-gp Inhibitor	AutoDock LBE (kcal/mol)	Predicted Inhibition Constant (µM)
Oxprenolol	−5.743 ± 0.398	70.273 ± 40.057
Promazine	−6.933 ± 0.021	8.273 ± 0.262
Riluzole	−5.380 ± 0.010	114.080 ± 2.326

**Table 11:** Average values of descriptors for inhibitors and substrates.

Descriptor	Inhibitor	Substrate
cLogP	3.498 ± 2.464	3.134 ± 2.962
Total surface area	311.199 ± 188.142	461.870 ± 286.187
Shape index	0.529 ± 0.125	0.429 ± 0.081
Molecular flexibility	0.395 ± 0.141	0.332 ± 0.114
Rotatable bonds	6.799 ± 12.158	9.818 ± 11.778
Aromatic rings	1.450 ± 1.168	1.918 ± 1.330
Aromatic atoms	8.237 ± 6.470	10.759 ± 7.098
Symmetric atoms	2.649 ± 3.637	3.582 ± 4.477
Aromatic nitrogens	0.301 ± 0.772	0.559 ± 1.141
Basic nitrogens	0.441 ± 0.625	0.659 ± 0.762
Acidic oxygens	0.117 ± 0.361	0.171 ± 0.462

## Supplementary Materials

The following are available online at https://www.mdpi.com/2073-4409/8/10/1286/s1, Table S1: Prediction of P-gp inhibitors identified by the random forest classification algorithm using the ChEMBL P-gp modulator list of 493 compounds, Table S2: Prediction of P-glycoprotein substrates identified by the RF classification algorithm using the ChEMBL P-gp modulator list of 150 compounds.

## Abbreviations

ABC	ATP binding cassette
AUC	area under the curve
kNN	k-nearest neighboring
MDR	multidrug resistance
P-gp	P-glycoprotein
RF	random forest
ROC	receiver operating characteristic
SVM	support vector machine

## References

[1] Efferth T., Zeino M., Volm M., Efferth T. (2015). Modulation of P-glycoprotein-mediated multidrug resistance by synthetic and phytochemical small molecules, monoclonal antibodies, and therapeutic nucleic acids. Resistance to Targeted ABC Transporters in Cancer.

[2] Krech T., Scheuerer E., Geffers R., Kreipe H., Lehmann U., Christgen M. (2012). ABCB1/MDR1 contributes to the anticancer drug-resistant phenotype of IPH-926 human lobular breast cancer cells. Cancer Lett..

[3] Burger H., Nooter K. (2004). Pharmacokinetic resistance to imatinib mesylate—Role of the ABC drug pumps ABCG2 (BCRP) and ABCB1 (MDR1) in the oral bioavailability of imatinib. Cell Cycle.

[4] Efferth T. (2009). Inhibition of P-glycoprotein at the blood brain barrier by phytochemicals derived from traditional Chinese medicine. Planta Medica.

[5] Kuete V., Fouotsa H., Mbaveng A.T., Wiench B., Nkengfack A.E., Efferth T. (2015). Cytotoxicity of a naturally occurring furoquinoline alkaloid and four acridone alkaloids towards multi-factorial drug-resistant cancer cells. Phytomedicine.

[6] Kadioglu O., Cao J., Kosyakova N., Mrasek K., Liehr T., Efferth T. (2016). Genomic and transcriptomic profiling of resistant CEM/ADR-5000 and sensitive CCRF-CEM leukaemia cells for unravelling the full complexity of multi-factorial multidrug resistance. Sci. Rep..

[7] Kuete V., Saeed M.E.M., Kadioglu O., Bortzler J., Khalid H., Greten H.J., Efferth T. (2015). Pharmacogenomic and molecular docking studies on the cytotoxicity of the natural steroid wortmannin against multidrug-resistant tumor cells. Phytomedicine.

[8] Efferth T., Osieka R. (1993). Clinical Relevance of the *Mdr-1* gene and its gene-product, P-glycoprotein for cancer chemotherapy—a metaanalysis. Tumordiagn Ther..

[9] Efferth T. (2001). The human ATP-binding cassette transporter genes: From the bench to the bedside. Curr. Mol. Med..

[10] Gillet J.P., Efferth T., Remacle J. (2007). Chemotherapy-induced resistance by ATP-binding cassette transporter genes. Biochim. Biophys. Acta Rev. Cancer.

[11] Kadioglu O., Saeed M.E.M., Valoti M., Frosini M., Sgaragli G., Efferth T. (2016). Interactions of human P-glycoprotein transport substrates and inhibitors at the drug binding domain: Functional and molecular docking analyses. Biochem. Pharmacol..

[12] Srinivas N.R. (2016). Understanding the role of tariquidar, a potent Pgp inhibitor, in combination trials with cytotoxic drugs: What is missing?. Cancer Chemother. Pharmacol..

[13] Kelly R.J., Draper D., Chen C.C., Robey R.W., Figg W.D., Piekarz R.L., Chen X., Gardner E.R., Balis F.M., Venkatesan A.M. (2011). A pharmacodynamic study of docetaxel in combination with the P-glycoprotein antagonist tariquidar (XR9576) in patients with lung, ovarian, and cervical cancer. Clin. Cancer Res..

[14] Abraham J., Edgerly M., Wilson R., Chen C., Rutt A., Bakke S., Robey R., Dwyer A., Goldspiel B., Balis F. (2009). A phase I study of the P-glycoprotein antagonist tariquidar in combination with vinorelbine. Clin. Cancer Res..

[15] Fox E., Widemann B.C., Pastakia D., Chen C.C., Yang S.X., Cole D., Balis F.M. (2015). Pharmacokinetic and pharmacodynamic study of tariquidar (XR9576), a P-glycoprotein inhibitor, in combination with doxorubicin, vinorelbine, or docetaxel in children and adolescents with refractory solid tumors. Cancer Chemother. Pharmacol..

[16] Zhang Y.M., Wang Y.C., Zhou W.N., Fan Y.R., Zhao J.N., Zhu L., Lu S., Lu T., Chen Y.D., Liu H.C. (2019). A combined drug discovery strategy based on machine learning and molecular docking. Chem. Boil. Drug Des..

[17] Zoffmann S., Vercruysse M., Benmansour F., Maunz A., Wolf L., Marti R.B., Heckel T., Ding H.Y., Truong H.H., Prummer M. (2019). Machine learning-powered antibiotics phenotypic drug discovery. Sci. Rep..

[18] Vamathevan J., Clark D., Czodrowski P., Dunham I., Ferran E., Lee G., Li B., Madabhushi A., Shah P., Spitzer M. (2019). Applications of machine learning in drug discovery and development. Nat. Rev. Drug Discov..

[19] Newman D.J., Cragg G.M. (2016). Natural Products as Sources of New Drugs from 1981 to 2014. J. Nat. Prod..

[20] Saeed M., Kadioglu O., Khalid H., Sugimoto Y., Efferth T. (2015). Activity of the dietary flavonoid, apigenin, against multidrug-resistant tumor cells as determined by pharmacogenomics and molecular docking. J. Nutr. Biochem..

[21] Seo E.J., Kuete V., Kadioglu O., Krusche B., Schroder S., Greten H.J., Arend J., Lee I.S., Efferth T. (2013). Antiangiogenic activity and pharmacogenomics of medicinal plants from traditional korean medicine. Evidence-Based Complement. Altern. Med..

[22] Broccatelli F. (2012). QSAR models for P-glycoprotein transport based on a highly consistent data set. J. Chem. Inf. Model..

[23] Zeino M., Saeed M.E.M., Kadioglu O., Efferth T. (2014). The ability of molecular docking to unravel the controversy and challenges related to P-glycoprotein-a well-known, yet poorly understood drug transporter. Investig. New Drugs.

[24] Sander T., Freyss J., von Korff M., Rufener C. (2015). Data Warrior: An open-source program for chemistry aware data visualization and analysis. J. Chem. Inf. Model..

[25] Lopez-Lopez E., Naveja J.J., Medina-Franco J.L. (2019). DataWarrior: An evaluation of the open-source drug discovery tool. Expert Opin. Drug Discov..

[26] Cai C.P., Fang J.S., Guo P.F., Wang Q., Hong H.X., Moslehi J., Cheng F.X. (2018). *In silico* pharmacoepidemiologic evaluation of drug-induced cardiovascular complications using combined classifiers. J. Chem. Inf. Model..

[27] Demsar J., Curk T., Erjavec A., Gorup C., Hocevar T., Milutinovic M., Mozina M., Polajnar M., Toplak M., Staric A. (2013). Orange: Data mining toolbox in python. J. Mach. Learn. Res..

[28] Alam A., Kowal J., Broude E., Roninson I., Locher K.P. (2019). Structural insight into substrate and inhibitor discrimination by human P-glycoprotein. Science.

[29] Lo Y.C., Rensi S.E., Torng W., Altman R.B. (2018). Machine learning in chemoinformatics and drug discovery. Drug Discov. Today.

[30] Zhang L., Tan J.J., Han D., Zhu H. (2017). From machine learning to deep learning: Progress in machine intelligence for rational drug discovery. Drug Discov. Today.

[31] Schierz A.C. (2009). Virtual screening of bioassay data. J. Cheminformatics.

[32] Cano G., Garcia-Rodriguez J., Garcia-Garcia A., Perez-Sanchez H., Benediktsson J.A., Thapa A., Barr A. (2017). Automatic selection of molecular descriptors using random forest: Application to drug discovery. Expert Syst. Appl..

[33] Ohashi R., Watanabe R., Esaki T., Taniguchi T., Torimoto-Katori N., Watanabe T., Ogasawara Y., Takahashi T., Tsukimoto M., Mizuguchi K. (2019). Development of simplified in vitro P-glycoprotein substrate assay and in silico prediction models to evaluate transport potential of P-glycoprotein. Mol. Pharm..

[34] Chen C., Lee M.H., Weng C.F., Leong M.K. (2018). Theoretical prediction of the complex P-glycoprotein substrate efflux based on the novel hierarchical support vector regression scheme. Molecules.

[35] Cui Y., Chen Q.G., Li Y.X., Tang L. (2017). A new model of flavonoids affinity towards P-glycoprotein: Genetic algorithm-support vector machine with features selected by a modified particle swarm optimization algorithm. Arch. Pharmacal. Res..

[36] Ngo T.D., Tran T.D., Le M.T., Thai K.M. (2016). Computational predictive models for P-glycoprotein inhibition of in-house chalcone derivatives and drug-bank compounds. Mol. Divers..

[37] Jiang L.D., Chen J.H., He Y.S., Zhang Y.L., Li G.Y. (2016). A method to predict different mechanisms for blood-brain barrier permeability of CNS activity compounds in Chinese herbs using support vector machine. J. Bioinform. Comput. Biol..

[38] Leong M.K., Chen H.B., Shih Y.H. (2012). Prediction of promiscuous P-glycoprotein inhibition using a novel machine learning scheme. PLoS ONE.

[39] Bikadi Z., Hazai I., Malik D., Jemnitz K., Veres Z., Hari P., Ni Z.L., Loo T.W., Clarke D.M., Hazai E. (2011). Predicting P-glycoprotein-mediated drug transport based on support vector machine and three-dimensional crystal structure of P-glycoprotein. PLoS ONE.

[40] Xue Y., Yap C.W., Sun L.Z., Cao Z.W., Wang J.F., Chen Y.Z. (2004). Prediction of P-glycoprotein substrates by a support vector machine approach. J. Chem. Inf. Comput. Sci..

[41] Keogh J.P., Kunta J.R. (2006). Development, validation and utility of an in vitro technique for assessment of potential clinical drug-drug interactions involving P-glycoprotein. Eur. J. Pharm. Sci..

[42] Lee W.Y., Cheung C.C., Liu K.W., Fung K.P., Wong J., Lai P.B., Yeung J.H. (2010). Cytotoxic effects of tanshinones from *Salvia miltiorrhiza* on doxorubicin-resistant human liver cancer cells. J. Nat. Prod..

[43] Takeshita H., Kusuzaki K., Ashihara T., Gebhardt M.C., Mankin H.J., Hirasawa Y. (1998). Actin organization associated with the expression of multidrug resistant phenotype in osteosarcoma cells and the effect of actin depolymerization on drug resistance. Cancer Lett..

[44] Silva R., Vilas-Boas V., Carmo H., Dinis-Oliveira R.J., Carvalho F., Bastos M.D., Remiao F. (2015). Modulation of P-glycoprotein efflux pump: Induction and activation as a therapeutic strategy. Pharmacol. Ther..

[45] Duarte N., Varga A., Cherepnev G., Radics R., Molnar J., Ferreira M.J.U. (2007). Apoptosis induction and modulation of P-glycoprotein mediated multidrug resistance by new macrocyclic lathyrane-type diterpenoids. Bioorganic Med. Chem..

[46] Medeiros B.C., Landau H.J., Morrow M., Lockerbie R.O., Pitts T., Eckhardt S.G. (2007). The farnesyl transferase inhibitor, tipifarnib, is a potent inhibitor of the *MDR1* gene product, P-glycoprotein, and demonstrates significant cytotoxic synergism against human leukemia cell lines. Leukemia.

[47] Rubin E.H., de Alwis D.P., Pouliquen I., Green L., Marder P., Lin Y., Musanti R., Grospe S.L., Smith S.L., Toppmeyer D.L. (2002). A phase I trial of a potent P-glycoprotein inhibitor, zosuquidar.3HCl trihydrochloride (LY335979), administered orally in combination with doxorubicin in patients with advanced malignancies. Clin. Cancer Res..

[48] Pauli-Magnus C., Murdter T., Godel A., Mettang T., Eichelbaum M., Klotz U., Fromm M.F. (2001). P-glycoprotein-mediated transport of digitoxin, alpha-methyldigoxin and beta-acetyldigoxin. Naunyn-Schmiedeberg’s Arch. Pharmacol..

[49] Vautier S., Lacomblez L., Chacun H., Picard V., Gimenez F., Farinotti R., Fernandez C. (2006). Interactions between the dopamine agonist, bromocriptine and the efflux protein, P-glycoprotein at the blood-brain barrier in the mouse. Eur. J. Pharm. Sci..

[50] Zhou L.J., Chen X.P., Gu Y.Q., Liang J.Y. (2009). Transport Characteristics of Candesartan in Human Intestinal Caco-2 Cell Line. Biopharmacy and Drug Disposal.

[51] Koizumi S., Konishi M., Ichihara T., Wada H., Matsukawa H., Goi K., Mizutani S. (1995). Flow Cytometric functional analysis of multidrug-resistance by Fluo-3—A comparison with rhodamine-123. Eur. J. Cancer.

[52] Zilfou J.T., Smith C.D. (1995). Differential interactions of cytochalasins with P-glycoprotein. Oncol. Res..

[53] Rebbeor J.F., Senior A.E. (1998). Effects of cardiovascular drugs on ATPase activity of P-glycoprotein in plasma membranes and in purified reconstituted form. Biophys. Acta (BBA) Biomembr..

[54] Yamazaki S., Costales C., Lazzaro S., Eatemadpour S., Kimoto E., Varma M.V. (2019). Physiologically-based pharmacokinetic modeling approach to predict rifampin-mediated intestinal P-glycoprotein induction. CPT: Pharmacometrics Syst. Pharmacol..

[55] Yasuda K., Lan L.B., Sanglard D., Furuya K., Schuetz J.D., Schuetz E.G. (2002). Interaction of cytochrome P450 3A inhibitors with P-glycoprotein. J. Pharmacol. Exp. Ther..

[56] Takeshita H., Kusuzaki K., Tsuji Y., Hirata M., Hashiguchi S., Nakamura S.I., Murata H., Ashihara T., Hirasawa Y. (1998). Avoidance of doxorubicin resistance in osteosarcoma cells using a new quinoline derivative, MS-209. Anticancer. Res..

[57] Luo F.R., Paranjpe P.V., Guo A., Rubin E., Sinko P. (2002). Intestinal transport of irinotecan in Caco-2 cells and MDCK II cells overexpressing efflux transporters PGP, cMOAT, and MRP1. Drug Metab. Dispos..

[58] Salvatore C.A., Moore E.L., Calamari A., Cook J.J., Michener M.S., O’Malley S., Miller P.J., Sur C., Williams D.L., Zeng Z.Z. (2010). Pharmacological properties of MK-3207, a potent and orally active calcitonin gene-related peptide receptor antagonist. J. Pharmacol. Exp. Ther..

[59] Nemcova-Furstova V., Kopperova D., Balusikova K., Ehrlichova M., Brynychova V., Vaclavikova R., Daniel P., Soucek P., Kovar J. (2016). Characterization of acquired paclitaxel resistance of breast cancer cells and involvement of ABC transporters. Toxicol. Appl. Pharmacol..

[60] Shepard R.L., Winter M.A., Hsaio S.C., Pearce H.L., Beck W.T., Dantzig A.H. (1998). Effect of modulators on the ATPase activity and vanadate nucleotide trapping of human P-glycoprotein. Biochem. Pharmacol..

[61] Eadie L.N., Hughes T.P., White D.L. (2016). ABCB1 Overexpression is a key initiator of resistance to tyrosine kinase inhibitors in CML cell lines. PLoS ONE.

[62] Nemethova V., Razga F. (2017). Overexpression of ABCB1 as prediction marker for CML: How close we are to translation into clinics?. Leukemia.

